# Supplementary material for: HIVprotI: an integrated web based platform for prediction and design of HIV proteins inhibitors
Source: J Cheminform. 2018 Mar 9;10:12. doi: 10.1186/s13321-018-0266-y (PMC5845081; doi:10.1186/s13321-018-0266-y)
Supplement: Supplementary file 2 — Additional file 2. Source code of HIVProtI web server. [file 13321_2018_266_MOESM2_ESM.zip › HIVprotI_Source-code/hivprotI/weka-3-6-6/doc/weka/clusterers/SimpleKMeans.html]

SimpleKMeans


---


|  |  |  |
| --- | --- | --- |
| **Overview** \| **Package** \| **Class** \| **Tree** \| **Deprecated** \| **Index** \| **Help** \| **Weka's home** | |  |
| **PREV CLASS**   **NEXT CLASS** | **FRAMES**    **NO FRAMES**     **All Classes** |  |
| SUMMARY: NESTED \| FIELD \| CONSTR \| METHOD | DETAIL: FIELD \| CONSTR \| METHOD |  |


---


## weka.clusterers Class SimpleKMeans

```
java.lang.Object
  weka.clusterers.AbstractClusterer
      weka.clusterers.RandomizableClusterer
          weka.clusterers.SimpleKMeans
```

**All Implemented Interfaces:**: java.io.Serializable, java.lang.Cloneable, Clusterer, NumberOfClustersRequestable, CapabilitiesHandler, OptionHandler, Randomizable, RevisionHandler, WeightedInstancesHandler

---

``` public class SimpleKMeans extends RandomizableClusterer implements NumberOfClustersRequestable, WeightedInstancesHandler ```

Cluster data using the k means algorithm


Valid options are:

```
 -N <num>
  number of clusters.
  (default 2).
```

```
 -V
  Display std. deviations for centroids.
```

```
 -M
  Replace missing values with mean/mode.
```

```
 -S <num>
  Random number seed.
  (default 10)
```

```
 -A <classname and options>
  Distance function to be used for instance comparison
  (default weka.core.EuclidianDistance)
```

```
 -I <num>
  Maximum number of iterations.
```

```
 -O 
  Preserve order of instances.
```

**Version:**
:   $Revision: 5538 $

**Author:**
:   Mark Hall (mhall@cs.waikato.ac.nz), Eibe Frank (eibe@cs.waikato.ac.nz)

**See Also:**: `RandomizableClusterer`, Serialized Form

---

**Constructor Summary**

`SimpleKMeans()`
  
          the default constructor


| **Method Summary** | |
| --- | --- |
| `void` | `buildClusterer(Instances data)`             Generates a clusterer. |
| `int` | `clusterInstance(Instance instance)`             Classifies a given instance. |
| `java.lang.String` | `displayStdDevsTipText()`             Returns the tip text for this property |
| `java.lang.String` | `distanceFunctionTipText()`             Returns the tip text for this property. |
| `java.lang.String` | `dontReplaceMissingValuesTipText()`             Returns the tip text for this property |
| `int[]` | `getAssignments()`             Gets the assignments for each instance |
| `Capabilities` | `getCapabilities()`             Returns default capabilities of the clusterer. |
| `Instances` | `getClusterCentroids()`             Gets the the cluster centroids |
| `int[][][]` | `getClusterNominalCounts()`             Returns for each cluster the frequency counts for the values of each nominal attribute |
| `int[]` | `getClusterSizes()`             Gets the number of instances in each cluster |
| `Instances` | `getClusterStandardDevs()`             Gets the standard deviations of the numeric attributes in each cluster |
| `boolean` | `getDisplayStdDevs()`             Gets whether standard deviations and nominal count Should be displayed in the clustering output |
| `DistanceFunction` | `getDistanceFunction()`             returns the distance function currently in use. |
| `boolean` | `getDontReplaceMissingValues()`             Gets whether missing values are to be replaced |
| `int` | `getMaxIterations()`             gets the number of maximum iterations to be executed |
| `int` | `getNumClusters()`             gets the number of clusters to generate |
| `java.lang.String[]` | `getOptions()`             Gets the current settings of SimpleKMeans |
| `boolean` | `getPreserveInstancesOrder()`             Gets whether order of instances must be preserved |
| `java.lang.String` | `getRevision()`             Returns the revision string. |
| `double` | `getSquaredError()`             Gets the squared error for all clusters |
| `java.lang.String` | `globalInfo()`             Returns a string describing this clusterer |
| `java.util.Enumeration` | `listOptions()`             Returns an enumeration describing the available options. |
| `static void` | `main(java.lang.String[] argv)`             Main method for testing this class. |
| `java.lang.String` | `maxIterationsTipText()`             Returns the tip text for this property |
| `int` | `numberOfClusters()`             Returns the number of clusters. |
| `java.lang.String` | `numClustersTipText()`             Returns the tip text for this property |
| `java.lang.String` | `preserveInstancesOrderTipText()`             Returns the tip text for this property |
| `void` | `setDisplayStdDevs(boolean stdD)`             Sets whether standard deviations and nominal count Should be displayed in the clustering output |
| `void` | `setDistanceFunction(DistanceFunction df)`             sets the distance function to use for instance comparison. |
| `void` | `setDontReplaceMissingValues(boolean r)`             Sets whether missing values are to be replaced |
| `void` | `setMaxIterations(int n)`             set the maximum number of iterations to be executed |
| `void` | `setNumClusters(int n)`             set the number of clusters to generate |
| `void` | `setOptions(java.lang.String[] options)`             Parses a given list of options. |
| `void` | `setPreserveInstancesOrder(boolean r)`             Sets whether order of instances must be preserved |
| `java.lang.String` | `toString()`             return a string describing this clusterer |

**Methods inherited from class weka.clusterers.RandomizableClusterer**

`getSeed, seedTipText, setSeed`

**Methods inherited from class weka.clusterers.AbstractClusterer**

`distributionForInstance, forName, makeCopies, makeCopy`

**Methods inherited from class java.lang.Object**

`equals, getClass, hashCode, notify, notifyAll, wait, wait, wait`

**Constructor Detail**

### SimpleKMeans

```
public SimpleKMeans()
```

:   the default constructor


**Method Detail**

### globalInfo

```
public java.lang.String globalInfo()
```

:   Returns a string describing this clusterer

    :   **Returns:**: a description of the evaluator suitable for displaying in the explorer/experimenter gui

---


### getCapabilities

```
public Capabilities getCapabilities()
```

:   Returns default capabilities of the clusterer.

    :   **Specified by:**: `getCapabilities` in interface `Clusterer` **Specified by:**: `getCapabilities` in interface `CapabilitiesHandler` **Overrides:**: `getCapabilities` in class `AbstractClusterer`
    :   **Returns:**: the capabilities of this clusterer **See Also:**: `Capabilities`

---


### buildClusterer

```
public void buildClusterer(Instances data)
                    throws java.lang.Exception
```

:   Generates a clusterer. Has to initialize all fields of the clusterer
    that are not being set via options.

    :   **Specified by:**: `buildClusterer` in interface `Clusterer` **Specified by:**: `buildClusterer` in class `AbstractClusterer`
    :   **Parameters:**: `data` - set of instances serving as training data **Throws:**: `java.lang.Exception` - if the clusterer has not been generated successfully

---


### clusterInstance

```
public int clusterInstance(Instance instance)
                    throws java.lang.Exception
```

:   Classifies a given instance.

    :   **Specified by:**: `clusterInstance` in interface `Clusterer` **Overrides:**: `clusterInstance` in class `AbstractClusterer`
    :   **Parameters:**: `instance` - the instance to be assigned to a cluster **Returns:**: the number of the assigned cluster as an interger if the class is enumerated, otherwise the predicted value **Throws:**: `java.lang.Exception` - if instance could not be classified successfully

---


### numberOfClusters

```
public int numberOfClusters()
                     throws java.lang.Exception
```

:   Returns the number of clusters.

    :   **Specified by:**: `numberOfClusters` in interface `Clusterer` **Specified by:**: `numberOfClusters` in class `AbstractClusterer`
    :   **Returns:**: the number of clusters generated for a training dataset. **Throws:**: `java.lang.Exception` - if number of clusters could not be returned successfully

---


### listOptions

```
public java.util.Enumeration listOptions()
```

:   Returns an enumeration describing the available options.

    :   **Specified by:**: `listOptions` in interface `OptionHandler` **Overrides:**: `listOptions` in class `RandomizableClusterer`
    :   **Returns:**: an enumeration of all the available options.

---


### numClustersTipText

```
public java.lang.String numClustersTipText()
```

:   Returns the tip text for this property

    :   **Returns:**: tip text for this property suitable for displaying in the explorer/experimenter gui

---


### setNumClusters

```
public void setNumClusters(int n)
                    throws java.lang.Exception
```

:   set the number of clusters to generate

    :   **Specified by:**: `setNumClusters` in interface `NumberOfClustersRequestable`
    :   **Parameters:**: `n` - the number of clusters to generate **Throws:**: `java.lang.Exception` - if number of clusters is negative

---


### getNumClusters

```
public int getNumClusters()
```

:   gets the number of clusters to generate

    :   **Returns:**: the number of clusters to generate

---


### maxIterationsTipText

```
public java.lang.String maxIterationsTipText()
```

:   Returns the tip text for this property

    :   **Returns:**: tip text for this property suitable for displaying in the explorer/experimenter gui

---


### setMaxIterations

```
public void setMaxIterations(int n)
                      throws java.lang.Exception
```

:   set the maximum number of iterations to be executed

    :   **Parameters:**: `n` - the maximum number of iterations **Throws:**: `java.lang.Exception` - if maximum number of iteration is smaller than 1

---


### getMaxIterations

```
public int getMaxIterations()
```

:   gets the number of maximum iterations to be executed

    :   **Returns:**: the number of clusters to generate

---


### displayStdDevsTipText

```
public java.lang.String displayStdDevsTipText()
```

:   Returns the tip text for this property

    :   **Returns:**: tip text for this property suitable for displaying in the explorer/experimenter gui

---


### setDisplayStdDevs

```
public void setDisplayStdDevs(boolean stdD)
```

:   Sets whether standard deviations and nominal count
    Should be displayed in the clustering output

    :   **Parameters:**: `stdD` - true if std. devs and counts should be displayed

---


### getDisplayStdDevs

```
public boolean getDisplayStdDevs()
```

:   Gets whether standard deviations and nominal count
    Should be displayed in the clustering output

    :   **Returns:**: true if std. devs and counts should be displayed

---


### dontReplaceMissingValuesTipText

```
public java.lang.String dontReplaceMissingValuesTipText()
```

:   Returns the tip text for this property

    :   **Returns:**: tip text for this property suitable for displaying in the explorer/experimenter gui

---


### setDontReplaceMissingValues

```
public void setDontReplaceMissingValues(boolean r)
```

:   Sets whether missing values are to be replaced

    :   **Parameters:**: `r` - true if missing values are to be replaced

---


### getDontReplaceMissingValues

```
public boolean getDontReplaceMissingValues()
```

:   Gets whether missing values are to be replaced

    :   **Returns:**: true if missing values are to be replaced

---


### distanceFunctionTipText

```
public java.lang.String distanceFunctionTipText()
```

:   Returns the tip text for this property.

    :   **Returns:**: tip text for this property suitable for displaying in the explorer/experimenter gui

---


### getDistanceFunction

```
public DistanceFunction getDistanceFunction()
```

:   returns the distance function currently in use.

    :   **Returns:**: the distance function

---


### setDistanceFunction

```
public void setDistanceFunction(DistanceFunction df)
                         throws java.lang.Exception
```

:   sets the distance function to use for instance comparison.

    :   **Parameters:**: `df` - the new distance function to use **Throws:**: `java.lang.Exception` - if instances cannot be processed

---


### preserveInstancesOrderTipText

```
public java.lang.String preserveInstancesOrderTipText()
```

:   Returns the tip text for this property

    :   **Returns:**: tip text for this property suitable for displaying in the explorer/experimenter gui

---


### setPreserveInstancesOrder

```
public void setPreserveInstancesOrder(boolean r)
```

:   Sets whether order of instances must be preserved

    :   **Parameters:**: `r` - true if missing values are to be replaced

---


### getPreserveInstancesOrder

```
public boolean getPreserveInstancesOrder()
```

:   Gets whether order of instances must be preserved

    :   **Returns:**: true if missing values are to be replaced

---


### setOptions

```
public void setOptions(java.lang.String[] options)
                throws java.lang.Exception
```

:   Parses a given list of options.


    Valid options are:

    ```
     -N <num>
      number of clusters.
      (default 2).
    ```

    ```
     -V
      Display std. deviations for centroids.
    ```

    ```
     -M
      Replace missing values with mean/mode.
    ```

    ```
     -S <num>
      Random number seed.
      (default 10)
    ```

    ```
     -A <classname and options>
      Distance function to be used for instance comparison
      (default weka.core.EuclidianDistance)
    ```

    ```
     -I <num>
      Maximum number of iterations.
    ```

    ```
     -O
      Preserve order of instances.
    ```

    :   **Specified by:**: `setOptions` in interface `OptionHandler` **Overrides:**: `setOptions` in class `RandomizableClusterer`
    :   **Parameters:**: `options` - the list of options as an array of strings **Throws:**: `java.lang.Exception` - if an option is not supported

---


### getOptions

```
public java.lang.String[] getOptions()
```

:   Gets the current settings of SimpleKMeans

    :   **Specified by:**: `getOptions` in interface `OptionHandler` **Overrides:**: `getOptions` in class `RandomizableClusterer`
    :   **Returns:**: an array of strings suitable for passing to setOptions()

---


### toString

```
public java.lang.String toString()
```

:   return a string describing this clusterer

    :   **Overrides:**: `toString` in class `java.lang.Object`
    :   **Returns:**: a description of the clusterer as a string

---


### getClusterCentroids

```
public Instances getClusterCentroids()
```

:   Gets the the cluster centroids

    :   **Returns:**: the cluster centroids

---


### getClusterStandardDevs

```
public Instances getClusterStandardDevs()
```

:   Gets the standard deviations of the numeric attributes in each cluster

    :   **Returns:**: the standard deviations of the numeric attributes in each cluster

---


### getClusterNominalCounts

```
public int[][][] getClusterNominalCounts()
```

:   Returns for each cluster the frequency counts for the values of each
    nominal attribute

    :   **Returns:**: the counts

---


### getSquaredError

```
public double getSquaredError()
```

:   Gets the squared error for all clusters

    :   **Returns:**: the squared error

---


### getClusterSizes

```
public int[] getClusterSizes()
```

:   Gets the number of instances in each cluster

    :   **Returns:**: The number of instances in each cluster

---


### getAssignments

```
public int[] getAssignments()
                     throws java.lang.Exception
```

:   Gets the assignments for each instance

    :   **Returns:**: Array of indexes of the centroid assigned to each instance **Throws:**: `java.lang.Exception` - if order of instances wasn't preserved or no assignments were made

---


### getRevision

```
public java.lang.String getRevision()
```

:   Returns the revision string.

    :   **Specified by:**: `getRevision` in interface `RevisionHandler` **Overrides:**: `getRevision` in class `AbstractClusterer`
    :   **Returns:**: the revision

---


### main

```
public static void main(java.lang.String[] argv)
```

:   Main method for testing this class.

    :   **Parameters:**: `argv` - should contain the following arguments: -t training file [-N number of clusters]


---


|  |  |  |
| --- | --- | --- |
| **Overview** \| **Package** \| **Class** \| **Tree** \| **Deprecated** \| **Index** \| **Help** \| **Weka's home** | |  |
| **PREV CLASS**   **NEXT CLASS** | **FRAMES**    **NO FRAMES**     **All Classes** |  |
| SUMMARY: NESTED \| FIELD \| CONSTR \| METHOD | DETAIL: FIELD \| CONSTR \| METHOD |  |


---
